# The Association of Systemic Microvascular Changes with Lung Function and Lung Density: A Cross-Sectional Study

**DOI:** 10.1371/journal.pone.0050224

**Published:** 2012-12-20

**Authors:** Bianca Harris, Ronald Klein, Michael Jerosch-Herold, Eric A. Hoffman, Firas S. Ahmed, David R. Jacobs, Barbara E. K. Klein, Tien Y. Wong, Joao A. C. Lima, Mary Frances Cotch, R. Graham Barr

**Affiliations:** 1 Department of Medicine, College of Physicians and Surgeons, Columbia University, New York, New York, United States of America; 2 Department of Ophthalmology and Visual Sciences, University of Wisconsin School of Medicine and Public Health, Madison, Wisconsin, United States of America; 3 Department of Radiology, School of Medicine, University of Minnesota, Minneapolis, Minnesota, United States of America; 4 Department of Radiology, Brigham and Women's Hospital, Harvard Medical School, Boston, Massachusetts, United States of America; 5 Department of Radiology, University of Iowa, Iowa City, Iowa, United States of America; 6 Department of Radiology, College of Physicians and Surgeons, Columbia University, New York, New York, United States of America; 7 Division of Epidemiology and Community Health, School of Public Health, University of Minnesota, Minneapolis, Minnesota, United States of America; 8 Department of Nutrition, University of Oslo, Olso, Norway; 9 Center for Eye Research Australia, University of Melbourne, Melbourne, Australia; 10 Singapore Eye Research Institute, National University of Singapore, Singapore, Singapore; 11 Departments of Medicine and Radiology, Johns Hopkins University, Baltimore, Maryland, United States of America; 12 Division of Epidemiology and Clinical Applications, National Eye Institute, National Institutes of Health, Bethesda, Maryland, United States of America; 13 Department of Epidemiology, Mailman School of Public Health, Columbia University, New York, New York, United States of America; University of Giessen Lung Center, Germany

## Abstract

Smoking causes endothelial dysfunction and systemic microvascular disease with resultant end-organ damage in the kidneys, eyes and heart. Little is known about microvascular changes in smoking-related lung disease. We tested if microvascular changes in the retina, kidneys and heart were associated with obstructive spirometry and low lung density on computed tomography. The Multi-Ethnic Study of Atherosclerosis recruited participants age 45–84 years without clinical cardiovascular disease. Measures of microvascular function included retinal arteriolar and venular caliber, urine albumin-to-creatinine ratio and, in a subset, myocardial blood flow on magnetic resonance imaging. Spirometry was measured following ATS/ERS guidelines. Low attenuation areas (LAA) were measured on lung fields of cardiac computed tomograms. Regression models adjusted for pulmonary and cardiac risk factors, medications and body size. Among 3,397 participants, retinal venular caliber was inversely associated with forced expiratory volume in one second (FEV_1_) (P<0.001) and FEV_1_/forced vital capacity (FVC) ratio (P = 0.04). Albumin-to-creatinine ratio was inversely associated with FEV_1_ (P = 0.002) but not FEV_1_/FVC. Myocardial blood flow (n = 126) was associated with lower FEV_1_ (P = 0.02), lower FEV_1_/FVC (P = 0.001) and greater percentage LAA (P = 0.04). Associations were of greater magnitude among smokers. Low lung function was associated with microvascular changes in the retina, kidneys and heart, and low lung density was associated with impaired myocardial microvascular perfusion. These cross-sectional results suggest that microvascular damage with end-organ dysfunction in all circulations may pertain to the lung, that lung dysfunction may contribute to systemic microvascular disease, or that there may be a shared predisposition.

## Introduction

Chronic obstructive pulmonary disease (COPD) recently surpassed stroke as the third leading cause of death in the United States [1]. Smoking is the major cause of COPD [2]; however, a minority of smokers develop COPD [3]. Cigarette smoke injures the airways and lung parenchyma by direct chemical exposure and inflammation induced predominantly via neutrophils, macrophages and T cells [4], [5].

Smoking is a major cause of endothelial dysfunction and microvascular disease throughout the body, caused in part by impairment of vascular endothelial growth factor (VEGF), with subsequent generation of reactive oxygen species and diminished nitric oxide release [6]. While it is known that smoking-related microvascular disease contributes to end-organ damage in the brain, kidney, heart and eyes [7], [8], it is unclear whether similar microvascular changes in the lung may contribute to COPD pathogenesis.

Animal studies suggest that endothelial dysfunction might contribute to COPD and emphysema [9], [10]. Several mechanisms have been proposed both *in vitro* and *in vivo* [11], [12], [13], [14]. Impaired flow mediated dilation (FMD) in large systemic arteries was associated with early COPD and emphysema [15],[16], as was impaired left ventricular filling [17] and increased pulmonary perfusion heterogeneity [18]. Endothelial microparticles reflective of endothelial apoptosis were increased in smokers with isolated reductions in diffusing capacity [19] and in COPD [20]. Together, these findings may suggest early pulmonary endothelial and microvascular damage in patients with COPD.

Whereas methods for assessing pulmonary microvascular compromise in the general population are limited, validated measures of systemic microvascular function are available for the retina, kidneys and heart. Retinal microvascular abnormalities include narrowing of retinal arterioles, predominantly from hypertension, and widening of retinal venules, predominantly from diabetes, inflammation and smoking. Both measures predict cardiovascular events in middle-aged individuals [21], notably in women (a 20 µm increase or decrease in retinal venular or arteriolar caliber, respectively, was associated increased risk of coronary heart disease) [22]. Microalbuminuria is a measure of renal microvascular damage that predicts cardiovascular events [23], as well as all-cause mortality in general population studies [24]. A two-fold increase in urine albumin excretion was associated with a relative risk of 1.29 for cardiovascular mortality, and 1.12 for overall mortality [25]. Myocardial blood flow (MBF) on magnetic resonance imaging (MRI) is a measure of cardiac microvascular perfusion, impairments in which can cause myocardial ischemia [26].

We examined the association of retinopathy, microalbuminuria and myocardial blood flow, respectively, with lung function and lung density on computed tomography (CT) in a large, multi-ethnic cohort free of clinical cardiovascular disease. We hypothesized that these measures of systemic microvascular changes were associated with reduced lung function and lower lung density, and that relationships would be of greater magnitude among smokers.

## Materials and Methods

### Study sample

The Multi-Ethnic Study of Atherosclerosis (MESA) is a multicenter prospective cohort study of white, African-American, Hispanic and Asian (predominantly of Chinese decent) adults [27]. In 2000–2002, MESA recruited 6,814 men and women ages 45–84 years old from six U.S. communities: Forsyth County, NC; Northern Manhattan and the Bronx, NY; Baltimore City and Baltimore County, MD; St. Paul, MN; Chicago, IL; and Los Angeles, CA. Exclusion criteria included clinical cardiovascular disease, weight greater than 300 lbs, pregnancy and impediment to long-term participation. All measures were ascertained at baseline except as noted below.

The MESA Lung Study enrolled 3,965 MESA participants of 4,484 selected who were sampled randomly among those who consented to genetic analyses, underwent baseline measures of endothelial function, and attended an examination during the MESA-Lung recruitment period in 2004–2006 (99%, 89%, and 91% of the MESA cohort, respectively). Asians were over-sampled.

Similar to prior studies [17],[28], we excluded a priori 322 participants with a restrictive pattern of spirometry, defined as a forced vital capacity (FVC) less than the lower limit of normal (LLN) [29], with a forced expiratory volume in one second (FEV_1_)/FVC ratio above the LLN, since the primary hypothesis related to obstructive lung disease.

### Ethics Statement

The protocols of MESA and all studies described herein were approved by the Institutional Review Boards of all collaborating institutions (Columbia University, New York, NY; Johns Hopkins University, Baltimore, MD; Northwestern University, Chicago, IL; University of California, Los Angeles, CA; University of Minnesota, Twin Cities, MN; Wake Forest University, Winston-Salem, NC) and the National Heart, Lung and Blood Institute (NHLBI). Written informed consent was obtained from all study participants.

### Microvascular Measures in the Retina, Kidney and Heart

#### Retinal Vascular Caliber

**Figure 1:**
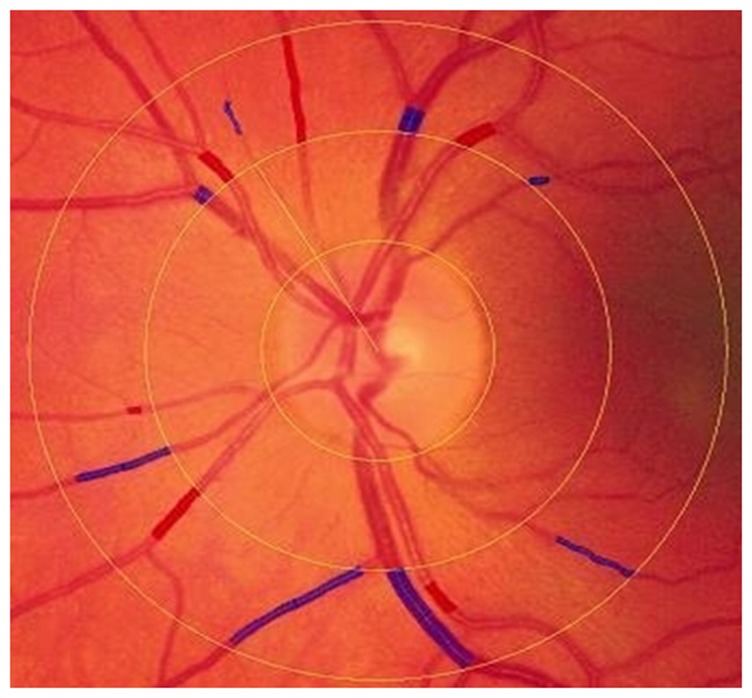
Digital photograph a MESA participant's retina showing central retinal vascular caliber. The caliber of retinal vessels is a calculated average of measurements of all arterioles and venules coursing through an area one half to one full disc diameter from the optic disc margin. Red shaded areas are used to compute the central retinal artery equivalent (CRAE) and blue shaded areas are used to compute the central retinal vein equivalent (CRVE).

Retinal vascular caliber was measured from digital retinal photographs of both eyes of each participant in 2002–03 [30]. All arterioles and venules coursing through an area one half to one full disc diameter from the optic disc margin (Figure 1) were measured using a computer-based program by trained graders masked to participant characteristics at a central reading center. Vascular caliber was summarized as the central retinal artery equivalent (CRAE) and the central retinal vein equivalent (CRVE), two well-established, reproducible indicators of the average caliber of retinal vessels [30].

#### Urine Albumin-to-Creatinine Ratio and Albuminuria

Urine albumin and creatinine were measured at the baseline examination by nephelometry and the rate Jaffe reaction. Spot urine albumin (µg/mL)-to-creatinine (µg/mL) ratios (ACRs) were calculated. Previously published, gender-specific categories of ACR were used to define albuminuria as high-normal urine albumin excretion, microalbuminuria and macroalbuminuria [31].

#### Myocardial Blood Flow

All participants at one field center were asked to participate in the myocardial perfusion study; 222 agreed and underwent the study, of whom 126 met inclusion criteria for the present report. MBF was measured using gadolinium-enhanced cardiac MRI (Figure S1) at rest and again during maximum adenosine-induced vasodilation (hyperemia) [32]. All imaging was performed on a 1.5 T magnet with a 2-coil anterior surface coil array positioned on the chest as a 2-coil phased array integrated in the patient bed for posterior signal reception. Image acquisition was gated electrocardiographically. MBF was determined by model-independent deconvolution and expressed as mL/g/min [33].

### Spirometry

Spirometry was conducted in 2004–2006 and in accordance with the American Thoracic Society/European Respiratory Society guidelines [34] using a dry-rolling-sealed spirometer with software that performed automated quality checks in real time (Occupational Marketing, Inc., Houston, TX). All spirometry exams were performed pre-bronchodilator and were reviewed by one investigator [35].

### Percent Low Attenuation Area

CT lung density was measured as percent lung low attenuation area (%LAA) on the lung fields of cardiac CT scans, which imaged approximately 70% of the lung volume from the carina to each lung base. Cardiac CT scans were performed at full inspiration on multi-detector (MD) and electron-beam CT scanners [36]. Two scans were performed on each participant; the scan with the higher air volume was used for analyses except in cases of discordant scan quality, in which case the higher quality scan was used.

Image attenuation was assessed using modified Pulmonary Analysis Software Suite [37] at a single reading center by trained readers without knowledge of other participant information. The attenuation of each pixel in the lung regions was linearly corrected, such that the mean attenuation outside the body was −1000 and aortic was 50 Hounsfield Units (HU). %LAA was defined as the percentage of the total voxels in the lung below −910 HU. This threshold was chosen based upon pathology comparisons [38] and the generally mild degree of emphysema in the sample. Sensitivity analyses were performed using %LAA defined as the percentage of the total voxels in the lung below −950 HU.

%LAA measures from the carina to lung base are highly correlated (r = 0.99) with full-lung measures on the same full-lung scans in smokers [39]. %LAA measures from cardiac scans correlated with those from full-lung scans from the same MESA participants [40] and have been used in this cohort to confirm multiple prior hypotheses [17], [28].

### Smoking

Cigarette, cigar and pipe smoking was self-reported using standardized questionnaire items [41]. Pack-years of cigarettes were calculated as age of starting to age of quitting or current age×(cigarettes-per-day/20). Cigarettes-per-day was assessed twice over a 4-year interval by questionnaire; the greater of the two measures were used in calculations. Current smoking was confirmed by urinary cotinine levels (Immulite 2000 Nicotine Metabolite Assay; Diagnostic Products Corp., Los Angeles, CA) [42].

### Covariates

Information on age, gender, race/ethnicity, educational attainment, occupational exposure to dust, fumes, or smoke, environmental tobacco smoke exposure, family history of emphysema, medical history and medication use were self-reported using standardized questionnaire items [41], [43], as recommended. Height, weight and resting blood pressure were measured using standard techniques, the latter using the Dinamap Monitor PRO 100 (Critikon, Tampa, FL). Serum glucose and lipids, including high- and low-density lipoproteins, were measured after 12-hour fast. The presence of diabetes mellitus was defined as fasting serum glucose level >126 mg/dL or current use of any diabetes medication.

### Statistical Analysis

The cohort was stratified by quartile of CRVE for descriptive purposes. Multivariate mean differences in lung function and %LAA were estimated using generalized linear models that regressed CRVE, CRAE, log-transformed ACR and MBF on spirometric and %LAA measurements after adjustment for age, gender, race/ethnicity, height, body mass index (BMI), waist and hip circumference. For CT analyses, models were also adjusted for scanner type and mAs. Multivariate models were then additionally adjusted for cigarette smoking status, cigarette pack years and urine cotinine, and subsequently the potential confounders listed in the footnotes to the tables. The presence of effect modification by smoking was tested with the – 2 log likelihood test of nested models with and without interaction terms for smoking. Analyses stratified by smoking status and restricted to individuals with airflow limitation as defined by FEV_1_/FVC<0.7 were also performed.

95% confidence intervals and P-values were estimated from generalized linear models. A P-value<0.05 was considered to indicate statistical significance. This threshold was not adjusted for multiple comparisons; rather, all analyses are reported, as recommended [44].

Statistical analyses were performed in SAS 9.2 (Cary, NC) and *R* Statistical Software, version 2.10.0 (R Foundation, Vienna, Austria) was used to generate graphs.

## Results

Figure S2 shows the recruitment of 3,965 participants in the MESA Lung Study and exclusions. There were no significant differences between participants with and without retinal and renal measures (data not shown) whereas those with MBF measures were younger, more often White or Hispanic, and more likely to report symptoms of chronic bronchitis (Table S1).

The overall sample of 3,643 was 61.1 years old at the baseline examination and 51% women. Fourteen percent were current smokers and 53% had ever smoked cigarettes. The mean FEV_1_ was 95.7% of predicted values and mean FEV_1_/FVC ratio was 74.5%.The median % LAA (<−910 HU) was 18.6 (interquartile range, IQR, 10.0, 29.3).

Table 1 shows the characteristics of the study sample by quartile of CRVE. Participants with a larger CRVE (4^th^ quartile) were more likely to be African-American or Hispanic, of younger age, higher BMI, and have lower education attainment. Tobacco exposure and diabetes were associated with larger CRVE, as were occupational exposure to dust and measures of inflammation.

**Table 1:** Characteristics of participants in the MESA Lung Study according to retinal vascular caliber as measured by central retinal vein equivalent (CRVE).

Characteristic	Quartile of Central Retinal Vein Equivalent			
	1^st^ Quartile 123.6–199.7	2^nd^ Quartile 199.7–214.2	3^rd^ Quartile 214.2–228.1	4^th^ Quartile 228.1–315.2
	N = 849	N = 849	N = 850	N = 849
Age, mean (SD), years	63.7 (10.0)	60.9 (9.6)	60.2 ( 9.5)	59.7 ( 9.6)
Male gender, %	47.4	50.1	49.4	49.7
Race/ethnicity, %				
*- White (Non-Hispanic)*	52.1	41.8	28.9	19.4
*- African American*	15.1	20.6	29.5	39.8
*- Asian*	15.2	16.5	17.1	16.6
*- Hispanic*	17.7	21.1	24.5	24.2
Education, years, %				
*- <High school*	12.5	14.4	18.6	19.7
*- High school & beyond*	87.5	67.6	81.4	80.3
Body mass index, mean (SD), kg/m^2^	27.2 (4.9)	27.7 (4.9)	28.2 (5.4)	28.7 (5.6)
Waist circumference, mean (SD), cm	95.6 (13.6)	96.3 (13.6)	97.5 (13.9)	98.5 (14.7)
Height, mean (SD), cm	166.3 (10.0)	167.0 (10.3)	166.3 (10.1)	166.7 (9.8)
Cigarette smoking,%				
*- Never*	51.5	52.5	46.1	39.2
*- Past*	41.1	38.2	38.9	36.4
*- Current*	7.4	9.3	15.1	24.4
Pack-years,* median (IQR)	15.0 (5.0, 33.0)	17.0 (6.0,32.3)	16.5 (6.3,34.2)	20.0 (7.6, 37.0)
Cotinine,† median (IQR), ng/mL	2351 (226, 6479)	3058 (769,6012)	3891 (1127,7039)	4864 (1594, 9203)
Environmental tobacco exposure, %	41.6	39.4	44.0	46.6
Family history of emphysema, %	5.2	3.7	6.4	3.9
Asthma before age 45 years, %	7.5	7.4	8.1	8.5
Hypertension, %	44.6	40.4	40.3	41.3
Blood pressure, mean (SD), mmHg				
*- Systolic*	125.2 (19.6)	124.1 (20.1)	124.1 (19.0)	124.2 (19.4)
*- Diastolic*	70.7 (10.2)	72.3 (10.1)	72.1 (9.7)	71.9 (9.8)
Diabetes mellitus, %	7.3	9.1	10.7	14.4
Fasting plasma glucose, median (IQR),mg/100 mL	95 (89,103)	95 (89,103)	97 (90,106)	98 (92,108)
High-density lipoprotein, mean (SD), mg/dL	53.6 (15.5)	51.4 (15.3)	50.4 (14.8)	48.3 (13.2)
Low-density lipoprotein, mean (SD), mg/dL	116.0 (29.8)	117.1 (30.7)	118.6 (30.9)	118.8 (31.8)

Abbreviations: SD = standard deviation; IQR = interquartile range.

Definitions: Hypertension = physician diagnosis of hypertension or systolic blood pressure >140 mmHg or diastolic blood pressure >90 mmHg; Diabetes mellitus = physician diagnosis of diabetes or fasting plasma glucose >126 mg/dl.

* Among ever smokers.

† Among current smokers.

### Retinal Vascular Caliber

**Figure 2:**
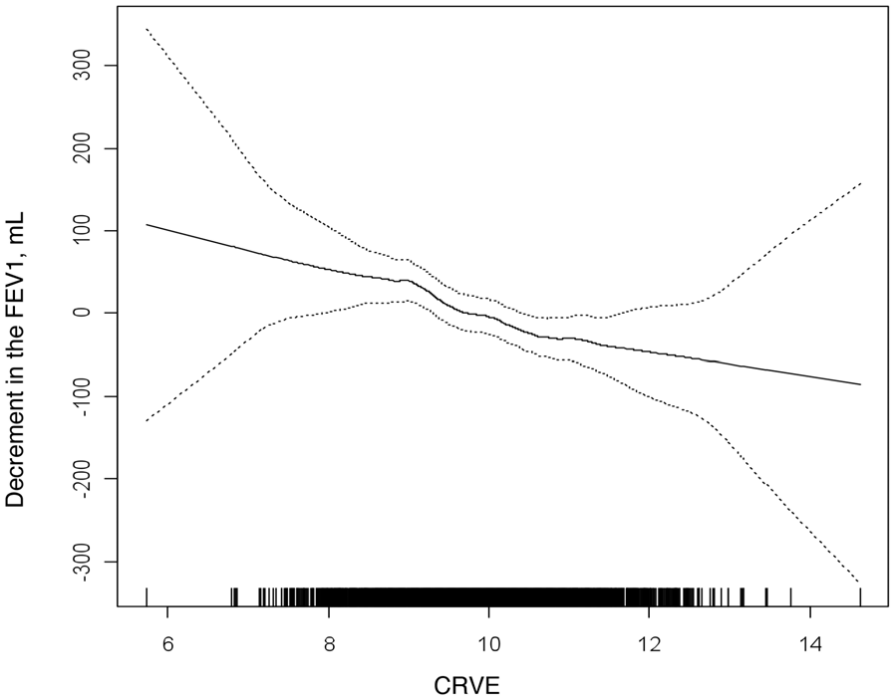
Multivariate association of the central retinal vein equivalent (CRVE) and the forced expiratory volume in one second (FEV_1_). The association of CRVE to the FEV_1_ was linear in the fully adjusted model, and without evidence for a threshold effect. Covariates include age, gender, race/ethnicity, height, BMI, waist and hip circumference, cigarette smoking status, pack-years, urine cotinine, cigar-years, pipe-years, environmental tobacco exposure, occupational exposure to dust, asthma before age 45, family history of emphysema, chronic bronchitis, educational attainment, diabetes mellitus, fasting blood glucose, hypertension, systolic blood pressure, diastolic blood pressure, high-density lipoprotein, low-density lipoprotein, C reactive protein, fibrinogen, aspirin use, beta blocker use, angiotensin II receptor blocker and/or angiotensin converting enzyme inhibitor use, statin use, diuretic use, hormone replacement therapy use, bronchodilator use, and oral or inhaled steroid use. Dotted lines are 95% confidence intervals.

Table 2 shows measures of lung function stratified by quartiles of CRVE and mean difference in lung function per standard deviation (SD) unit of CRVE. There were highly significant associations between CRVE and all spirometric measures. These associations were somewhat attenuated after adjustment for smoking variables but remained statistically significant after adjustment for multiple measures of smoking in addition to other potential confounders. The association of CRVE to the FEV_1_ was linear without evidence for a threshold effect (Figure 2).

**Table 2:** Mean differences in lung function and percent low attenuation area (%LAA) by retinal vascular caliber as measured by central retinal vein equivalent (CRVE).

	Quartile of Central Retinal Vein Equivalent				Mean difference per 1 SD unit of CRVE (95% CI)	P-value
	1^st^ Quartile 123.6–199.7	2^nd^ Quartile 199.7–214.2	3^rd^ Quartile 214.2–228.1	4^th^ Quartile 228.1–315.2		
	N = 849	N = 849	N = 850	N = 849		
**FEV_1_,mL**						
Model 1†	0	−37	−78	−129	−53 (−69,−38)	<0.001
Model 2‡	0	−31	−55	−81	−33 (−48,−18)	<0.001
Model 3§	0	−35	−46	−71	−28 (−43,−14)	<0.001
**FEV_1/_FVC,%** *						
Model 1†	0	−0.9	−1.5	−1.9	−0.8 (−1.0,−0.5)	<0.001
Model 2‡	0	−0.8	−1.0	−0.9	−0.3 (−0.6,−0.1)	0.02
Model 3§	0	−0.9	−0.9	−0.8	−0.3 (−0.6,−0.0)	0.04
**LAA, %**						
Model 1†	0	−1.1	−1.4	−1.6	−0.6 (−1.0,−0.2)	0.004
Model 2‡	0	−1.1	−1.2	−1.0	−0.3 (−0.8, 0.1)	0.11
Model 3§	0	−1.1	−1.1	−1.0	−0.3 (−0.8, 0.1)	0.13

Abbreviations: SD = standard deviation; CRVE = Central Retinal Vein Equivalent; CI = confidence interval.

* Includes 16 fewer participants than FEV_1_ analysis.

† Model 1: Adjusted for age, gender, race/ethnicity, body mass index, height, waist and hip circumference and, for CT analyses, CT scanner type.

‡ Model 2: Adjusted for all the variables in model 1 plus cigarette smoking status, cigarette pack years and urine cotinine.

§ Model 3: Adjusted for all the variables in model 2 plus cigar-years, pipe-years, environmental tobacco exposure, occupational exposure to dust, asthma before age 45, family history of emphysema, chronic bronchitis, educational attainment, diabetes mellitus, fasting blood glucose, hypertension, systolic blood pressure, diastolic blood pressure, high-density lipoprotein, low-density lipoprotein, C reactive protein, fibrinogen, aspirin use, beta blocker use, angiotensin II receptor blocker and/or angiotensin converting enzyme inhibitor use, statin use, diuretic use, hormone replacement therapy use, bronchodilator use, oral or inhaled steroid use.

The relationship of CRVE with the FEV_1_ was modified by smoking status (P-interaction = 0.008): a 93 mL decrement in current smokers (95% CI {−148, −38}; P<0.001), 48 mL decrement in former smokers (95% CI {−73, −23}; p<0.001), and 4 mL decrement in never smokers per SD unit of CRVE (95% CI {−23, 15}; P = 0.67).

There was no evidence that CRVE was associated with %LAA after adjustment for smoking variables and other potential confounders. CRAE was associated with neither lung function nor %LAA (Table S2).

### Albuminuria

**Figure 3:**
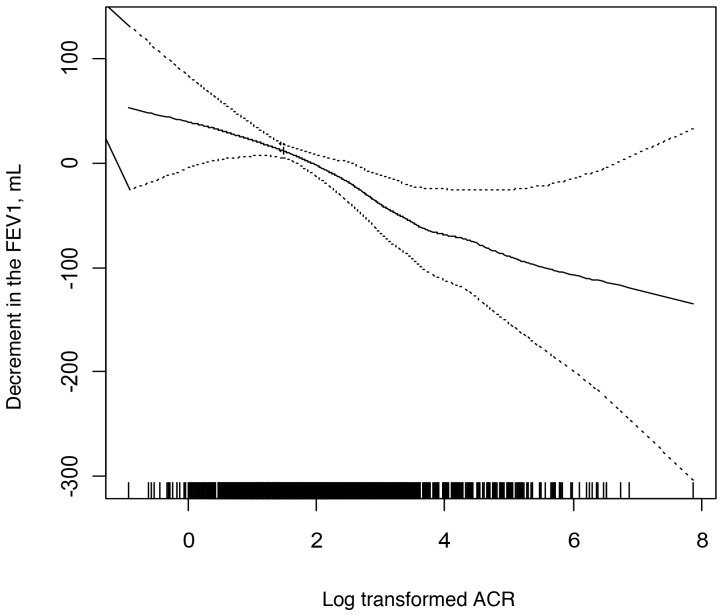
Multivariate association of the log-transformed albumin-to-creatinine ratio (ACR) and the forced expiratory volume in one second (FEV_1_). The association of ACR to FEV_1_ was linear on a log scale in the fully adjusted multivariate model. Covariates include age, gender, race/ethnicity, height, BMI, waist and hip circumference, cigarette smoking status, pack-years, urine cotinine, cigar-years, pipe-years, environmental tobacco exposure, occupational exposure to dust, asthma before age 45, family history of emphysema, chronic bronchitis, educational attainment, diabetes mellitus, fasting blood glucose, hypertension, systolic blood pressure, diastolic blood pressure, high-density lipoprotein, low-density lipoprotein, C reactive protein, fibrinogen, aspirin use, beta blocker use, angiotensin II receptor blocker and/or angiotensin converting enzyme inhibitor use, statin use, diuretic use, hormone replacement therapy use, bronchodilator use, and oral or inhaled steroid use. Dotted lines are 95% confidence intervals.

Table 3 shows measures of lung function and %LAA by urine albumin excretion (UAE) as defined by degree of albuminuria. Mean difference in FEV_1_ was −25 mL per SD unit of log-transformed ACR (95% CI {−40, −9}, P = 0.002).There were highly significant associations of UAE and ACR, respectively, to the FEV_1_ and FVC before and after adjustment for smoking variables and other covariates, but not to the FEV_1_/FVC ratio. The association of ACR to the FEV_1_ was linear on a log scale (Figure 3).

**Table 3:** Mean differences in lung function and percent low attenuation area (%LAA) by renal function, as measured by urine albumin excretion (UAE) categories defined by albumin-to-creatinine ratio (ACR).

	Urine Albumin Excretion				P-value
	None	High normal	Micro-albuminuria	Macro-albuminuria	
	N = 2802	N = 396	N = 394	N = 35	
**FEV_1_,mL**					
Model 1†	0	−79	−106	−109	<0.0001
Model 2‡	0	−80	−97	−108	0.0002
Model 3§	0	−58	−70	−49	0.0006
**FEV_1/_FVC,%** *					
Model 1†	0	−0.6	−0.7	1.1	0.18
Model 2‡	0	−0.6	−0.5	1.1	0.65
Model 3§	0	−0.5	−0.7	0.5	0.12
**LAA, %**					
Model 1†	0	−0.3	−2.1	−1.2	0.0022
Model 2‡	0	−0.4	−2.1	−1.5	0.24
Model 3§	0	−0.1	−1.3	−0.3	0.11

Abbreviations: SD = standard deviation; ACR = albumin-to-creatinine ratio; CI = confidence interval.

Definitions: 1) High normal urine albumin excretion: ACR 9–16.9 mg/g in men and 13–24.9 mg/g in women; 2) Microalbuminuria: ACR 17–250 mg/g in men and 25–354.9 mg/g in women; and 3) Macroalbuminuria: ACR ≥250 mg/g in men and ≥355 mg/g in women.

* Includes 17 fewer participants than FEV_1_ analysis.

† Model 1: Adjusted for age, gender, race/ethnicity, body mass index, height, waist and hip circumference and, for CT analyses, CT scanner type.

‡ Model 2: Adjusted for all the variables in model 1 plus cigarette smoking status, cigarette pack years and urine cotinine.

§ Model 3: Adjusted for all the variables in model 2 plus cigar-years, pipe-years, environmental tobacco exposure, occupational exposure to dust, asthma before age 45, family history of emphysema, chronic bronchitis, educational attainment, diabetes mellitus, fasting blood glucose, hypertension, systolic blood pressure, diastolic blood pressure, high-density lipoprotein, low-density lipoprotein, C reactive protein, fibrinogen, aspirin use, beta blocker use, angiotensin II receptor blocker and/or angiotensin converting enzyme inhibitor use, statin use, diuretic use, hormone replacement therapy use, bronchodilator use, oral or inhaled steroid use.

Smoking status did not modify the associations of ACR with lung function (P-interaction>0.10) although the magnitude of the association was greater in smokers: a decrement in the FEV_1_ of 67 mL (95% CI {−121, −13}; P = 0.02) in current smokers, 48 mL (95% CI {−71, −26}; P<0.001) in former smokers and 18 ml (95% CI { −36, 0.1}; P = 0.06) in never smokers.

Associations with %LAA were not statistically significant in fully adjusted models for UAE (Table 3) or for ACR (−0.3% per 1 SD unit of ACR, 95% CI {−0.8, 0.1}; P = 0.13).

### Myocardial Perfusion

Mean differences in lung function and %LAA per SD unit of resting and hyperemic MBF are shown in Table 4. In fully adjusted models, hyperemic MBF was positively associated with measures of airflow limitation (both FEV_1_ and FEV_1_/FVC) and inversely associated with %LAA. Findings were essentially unchanged after additional adjustment of hyperemic MBF for resting MBF (FEV_1_ 112 mL, 95% CI {14, 210}; P-value 0.03; FEV_1_/FVC 2.9%, 95% CI {1.1, 4.6}; P = 0.002; and %LAA −2.6%, 95% CI {−5.5, 0.2}; P = 0.07). In addition, %LAA was associated with resting MBF. Associations were qualitatively of greater magnitude among smokers (data not shown).

**Table 4:** Mean differences in lung function and percent low attenuation area (%LAA) by myocardial blood flow (MBF) at rest and during hyperemia.

	Resting MBF, ml/g/min (95% CI)	P-value	Hyperemic MBF, ml/g/min (95% CI)	P-value
**FEV_1_,mL**				
Model 1†	150 (−226, 526)	0.43	62 (−47, 171)	0.26
Model 2‡	112 (−245, 469)	0.54	94 (−8, 197)	0.07
Model 3§	118 (−244, 481)	0.52	113 (16, 211)	0.02
**FEV_1/_FVC,%**				
Model 1†	5.7 (−0.6, 11.9)	0.08	2.0 (−0.2, 3.8)	0.03
Model 2‡	4.3 (−1.5, 10.0)	0.14	2.5 (0.8, 4.1)	0.003
Model 3§	5.1 (−1.7, 11.8)	0.14	3.0 (1.2, 4.7)	0.001
**LAA, %**				
Model 1†	−9.4 (−19.6, 0.9)	0.07	−1.7 (−4.6, 1.3)	0.27
Model 2‡	−8.1 (−17.7, 1.5)	0.10	−1.4 (−4.2, 1.4)	0.34
Model 3§	−14.7 (−25.4, −3.9)	0.008	−3.1 (−6.0, 0.1)	0.04

Abbreviations: SD = standard deviation; MBF = myocardial blood flow; CI = confidence interval;

† Model 1: Adjusted for age, gender, race/ethnicity, body mass index, height, waist and hip circumference and, for CT analyses, CT scanner type.

‡ Model 2: Adjusted for all the variables in model 1 plus cigarette smoking status, cigarette pack years and urine cotinine.

§ Model 3: Adjusted for all the variables in model 2 plus cigar-years, pipe-years, environmental tobacco exposure, occupational exposure to dust, asthma before age 45, family history of emphysema, chronic bronchitis, educational attainment, diabetes mellitus, fasting blood glucose, hypertension, systolic blood pressure, diastolic blood pressure, heart rate, high-density lipoprotein, low-density lipoprotein, C reactive protein, fibrinogen, aspirin use, beta blocker use, angiotensin II receptor blocker and/or angiotensin converting enzyme inhibitor use, statin use, diuretic use, hormone replacement therapy use, bronchodilator use, oral or inhaled steroid use.

### Supplemental Analyses

After the exclusion of participants with diabetes, the magnitude of associations for the FEV_1_ and FEV_1_/FVC ratio generally increased. Exclusion of participants with hypertension had little impact on the results and there was no evidence for effect modification by race/ethnicity or gender (data not shown).

The relationship between CRVE and FEV_1_/FVC was similar among 783 participants with airflow limitation compared to all participants but did not attain statistical significance (−0.02% per 1 SD unit CRVE, P = 0.06). The association between FEV_1_ and ACR among those with airflow limitation was attenuated (a decrement in FEV_1_ of 13 mL per 1 SD unit ACR; P = 0.58).

Using a threshold of −950 HU to define %LAA, the association with resting MBF remained significant (P = 0.004), the relationship with hyperemic MBF did not (P = 0.12), and the statistical significance of all other results were unchanged (data not shown). Sensitivity analysis using an inflated measure of pack-years also did not alter the results.

## Discussion

Microvascular changes in the retina, kidneys and heart were associated with decrements in lung function in this large, population-based cohort free of clinical cardiovascular disease. In addition, impaired myocardial microvascular function on MRI scanning was associated with lower lung function and lower lung density on CT scan.

We assessed three complementary measures of systemic microvascular disease in the retinal, renal and myocardial circulations. The first, retinal vascular caliber, has been widely used as a marker for changes in the microvasculature [45], [46]. No prior studies have examined retinal vascular caliber in COPD, to our knowledge, although one study found impaired hemodynamic changes of extraocular orbital arteries in COPD patients compared to healthy controls [47].

Retinal venular caliber, which is typically considered reflective of early diabetic microvascular changes [46], was linearly related to an obstructive pattern of spirometry. In studies of the effect of hyperglycemia on retinal blood flow, venular vasodilation is thought to occur due to oxidative stress and local “pseudohypoxia” [48]. In addition, endothelial dysfunction, abnormal nitric oxide levels and inflammatory mediators interfere with vascular auto-regulation, leading to vasodilation of the retinal veins [49]. Retinal venular caliber in MESA was associated with brachial artery FMD, a measure of endothelial dysfunction [50], and we have previously shown that brachial artery FMD is associated with lung function and %LAA [15]. The observed association of CVRE with lung function may therefore reflect endothelial dysfunction or hypoxemia, although it is unlikely that many participants in this population-based study were significantly hypoxemic.

In contrast, there was no evidence for an association of lung function with retinal arteriolar caliber, which is typically narrowed in hypertension and hypertensive arteriopathies [30].

The second microvascular measure, albuminuria, reflects endothelial dysfunction and microvascular damage in the renal circulation [51]. A prior small study found a significant relationship between microalbuminuria and COPD exacerbations [52], and in another, microalbuminuria was greater among 129 COPD patients compared to 51 controls [53]. The lack of association in the present study between albuminuria and FEV_1_/FVC may reflect residual confounding by obesity. Although we did not detect an association with low lung density in this healthy cohort, severe emphysema, as measured by %LAA on quantitative CT, was recently shown to be associated with reduced glomerular filtration rate in smokers [54].

The third measure, myocardial perfusion, is thought to be affected by impairment of smooth muscle relaxation and endothelial dysfunction at a microcirculatory level in the absence of coronary epicardial stenosis [32]. In the present study, coronary vasodilatation with adenosine demonstrated an association of hyperemic MBF with lung function, which suggests impaired cardiac microvascular perfusion. The relationship of resting MBF to %LAA also suggests impairment of microvascular blood flow in the heart. This association is unlikely to reflect any epicardial abnormalities since there is no evidence for an association of %LAA with coronary artery calcium in this cohort [28]. A previous thallium-201 imaging study reported reversible perfusion defects in COPD patients attributable to reduced thallium uptake due to cellular dysfunction [55]. The present study adds to this finding by linking the reduced contrast uptake to microvascular dysfunction, as gadolinium is an extracellular, blood-borne tracer that allows direct estimation of MBF and microvascular function without regard to cellular function.

Taken in totality, findings from this large study with a unique combination of microvascular measures suggest that there are diffuse systemic microvascular changes associated with decrements in lung function in healthy individuals, especially in smokers. While the cross-sectional design of the study prevents definitive conclusions about causality and the direction of the association (i.e. whether microvascular changes contribute to airflow limitation or the reverse), we speculate smoking could cause microvascular disease with resultant end-organ damage to the lungs via related mechanisms by which it causes microvascular disease in the systemic circulation. Although this concept is relatively unexplored in COPD as classically defined by airflow obstruction, this mechanism is biologically plausible given the proximal dose of tobacco smoke to the pulmonary circulation and the small probability that the pulmonary microcirculation is immune to the effects of tobacco smoke.

The few existing biopsy studies on pulmonary microvascular structure in COPD demonstrate microvascular remodeling and redistribution [56], [57], [58], [59] and functional studies show that airway blood flow and vasodilator response is reduced in COPD [60]. In a mouse model, tobacco smoke-induced alterations in lung vascular structure and function preceded emphysema, and were independent of hypoxia [61].

Alternatively, decrements in lung function and structure might cause systemic microvascular disease, potentially via hypoxemia [53], which was not measured in the present study. Another possibility is a shared susceptibility to cigarette smoke; that is, persons with a susceptibility to smoking-related systemic microvascular disease are also susceptible to smoking-related lung disease. For example, genome-wide association studies of lung function have found significant associations for variants in the receptor for advanced glycation end products (RAGE) gene, which is also implicated in microvascular disease [62].

There are several limitations to our study. There is the potential for residual confounding in observational studies, particularly by smoking. However, dose of current smoking was precisely measured by the assessment of cotinine levels and sensitivity analyses using inflated measures of pack-years yielded similar results (data not shown). An additional limitation is that %LAA was assessed on cardiac scans, an approach which has been previously validated in this cohort and which has confirmed prior hypotheses [17], [28], [40]. Smoking-related emphysema has an apical predominance and the apices were less accurately measured than the bases in the current study because the coronary calcium scans assessed the lower 70% of the lung. This may explain why associations of the retina and kidney were more evident for measures of lung function than lung density, which is the opposite of what we found in a study of endothelial dysfunction among smokers using full-lung scans [15]. Finally, spirometry was measured approximately four years after the other measures, which could have resulted in temporally dissociated relationships; however, this is unlikely to have had a significant impact on the results given the small change in spirometric values over this time period. Despite these limitations, the large number of participants, including 783 with airflow limitation, contributes to both the power and precision of findings.

In conclusion, low lung function was associated with microvascular changes in the retina, kidney and heart, and low lung density was associated with impaired myocardial microvascular perfusion. These findings suggest that obstructive decrements in lung function may be characterized by defects in the microvascular circulation, which may provide an important link between cardiovascular and pulmonary disease.

## Supporting Information

Figure S1 Measurement of myocardial blood flow (MBF) using magnetic resonance imaging (MRI).**Figure S1a.** The three MRI images from a MESA participant show selected phases for measurement of MBF depicting 1) the transit of a contrast bolus in the right ventricle, 2) peak enhancement of the left ventricular blood pool, and 3) maximum myocardial enhancement. **Figure S1b–d.** Signal intensity versus time curves were generated in a region of the left ventricle (Fig. S1b) and the anterior myocardial sector (Fig. S1d). MBF was determined by optimizing the shape of the maximum tissue impulse response (Fig. S1c) in conjunction with arterial input (convolution operator symbolized by ∶ in Fig. S1b). The line of best fit is shown in Fig. S1d.
(TIF)

Figure S2 Recruitment of the MESA Lung Study and exclusions for the current study sample. 3,965 participants were recruited from the overall MESA cohort to the MESA Lung Study. Subsequent exclusions based on available/acceptable measurements and restriction on spirometry yielded 3,397 participants in the retinal analysis and 3,627 participants in the renal analysis. Given the availability myocardial blood flow (MBF) measurements in a subset of MESA-Lung participants at one study site, there were 126 participants available for cardiac analysis.
(TIF)

Table S1 Comparison of characteristics of participants in overall sample with and without myocardial perfusion measurements.
(DOCX)

Table S2 Lung function and percent low attenuation area (%LAA) by retinal vascular caliber as measured by central retinal artery equivalent (CRAE).
(DOCX)
