# Supplementary figures and images for: The Association of Systemic Microvascular Changes with Lung Function and Lung Density: A Cross-Sectional Study
Source: PLoS One. 2012 Dec 20;7(12):e50224. doi: 10.1371/journal.pone.0050224 (PMC3527439; doi:10.1371/journal.pone.0050224)

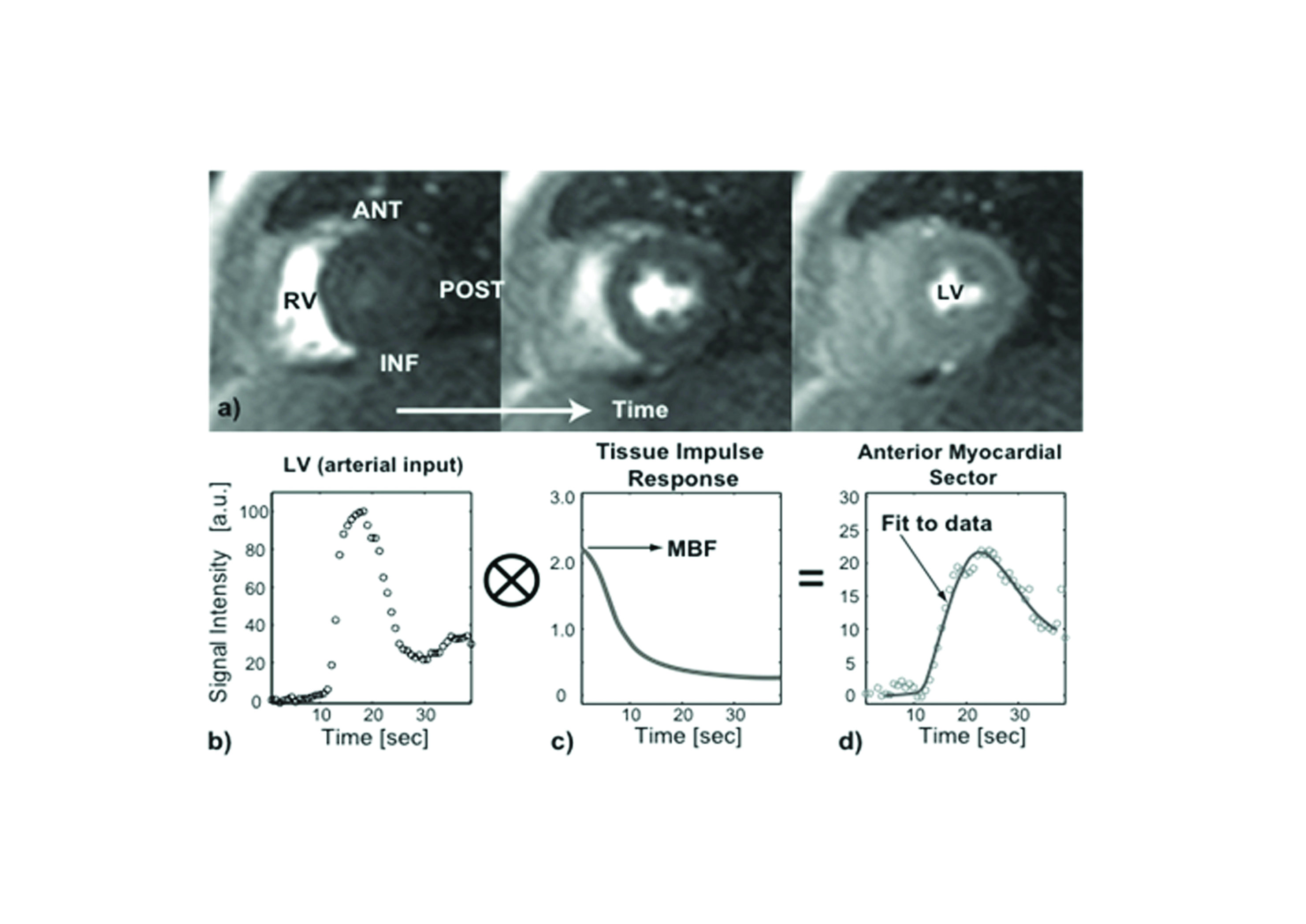

Supplement: Figure S1 — Measurement of myocardial blood flow (MBF) using magnetic resonance imaging (MRI).Figure S1a. The three MRI images from a MESA participant show selected phases for measurement of MBF depicting 1) the transit of a contrast bolus in the right ventricle, 2) peak enhancement of the left ventricular blood pool, and 3) maximum myocardial enhancement. Figure S1b–d. Signal intensity versus time curves were generated in a region of the left ventricle (Fig. S1b) and the anterior myocardial sector (Fig. S1d). MBF was determined by optimizing the shape of the maximum tissue impulse response (Fig. S1c) in conjunction with arterial input (convolution operator symbolized by ∶ in Fig. S1b). The line of best fit is shown in Fig. S1d. (TIF) [file pone.0050224.s001.tif]

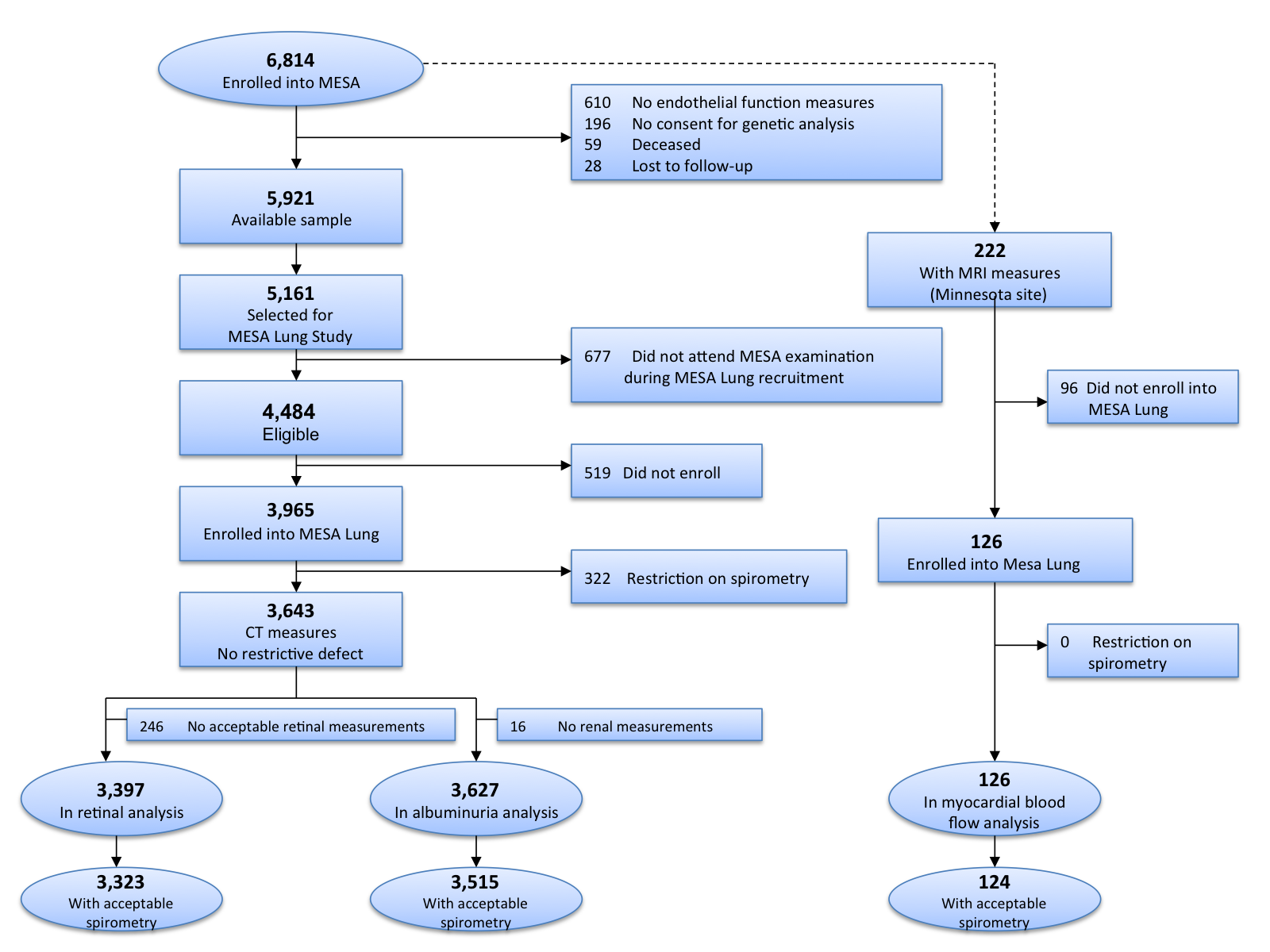

Supplement: Figure S2 — Recruitment of the MESA Lung Study and exclusions for the current study sample. 3,965 participants were recruited from the overall MESA cohort to the MESA Lung Study. Subsequent exclusions based on available/acceptable measurements and restriction on spirometry yielded 3,397 participants in the retinal analysis and 3,627 participants in the renal analysis. Given the availability myocardial blood flow (MBF) measurements in a subset of MESA-Lung participants at one study site, there were 126 participants available for cardiac analysis. (TIF) [file pone.0050224.s002.tif]
