# Supplementary material for: The Association of Systemic Microvascular Changes with Lung Function and Lung Density: A Cross-Sectional Study
Source: PLoS One. 2012 Dec 20;7(12):e50224. doi: 10.1371/journal.pone.0050224 (PMC3527439; doi:10.1371/journal.pone.0050224)
Supplement: Table S1 — Comparison of characteristics of participants in overall sample with and without myocardial perfusion measurements. (DOCX) [file pone.0050224.s003.docx]

**Table S1**. **Comparison of characteristics of participants in overall sample with and without myocardial perfusion measurements.**

| **Characteristic** | **Without** | **With** | **P-value** |
| --- | --- | --- | --- |
|  | N= 3397 | N=126 |  |
| Age (mean), years | 61.6 | 58.9 | 0.003 |
| Male gender, % | 49 | 56 | 0.09 |
| Race/Ethnicity, % |  |  |  |
| *- White (Non-Hispanic)* | 34 | 57 | <0.0001 |
| - *Hispanic* | 21 | 43 |  |
| *- Asian* | 17 | 0 |  |
| *- African-American* | 27 | 0 |  |
| Education, % |  |  |  |
| *- < High school* | 17 | 10 | 0.008 |
| *- High school and beyond* | 83 | 90 |  |
| Body mass index (mean), kg/m^2^ | 27.9 | 28.6 | 0.08 |
| Waist circumference (mean), cm | 97 | 98 | 0.24 |
| Height (mean), cm | 166 | 168 | 0.08 |
| Cigarette Smoking Status, % |  |  |  |
| *- Never* | 48 | 37 | 0.04 |
| *- Former* | 38 | 49 |  |
| *- Current* | 14 | 14 |  |
| Pack-Years (median)* | 17 | 19 | 0.78 |
| Cotinine † (median), ng/mL | 3789 | 4754 | 0.90 |
| Environmental tobacco exposure, % | 43 | 42 | 0.90 |
| Family History of Emphysema, % | 5 | 3 | 0.42 |
| Chronic bronchitis, % | 8 | 18 | <.0001 |
| Asthma before age 45 , % | 8 | 4 | 0.10 |
| Hypertension, % | 43 | 23 | <.0001 |
| Blood pressure (mean), mmHg |  |  |  |
| *- Systolic* | 125 | 119 | 0.0003 |
| *- Diastolic* | 72 | 70 | 0.08 |
| Diabetes mellitus, % | 28 | 14 | 0.08 |
| Fasting glucose (median), mg/100mL | 96.0 | 94.5 | 0.002 |
| High-density lipoprotein (mean), mg/dL | 51 | 48 | 0.008 |
| Low-density lipoprotein (mean), mg/dL | 117 | 121 | 0.25 |

Definitions: Chronic bronchitis = chronic productive cough in 3 or more months in 2 or more years; hypertension = physician diagnosis of hypertension or systolic blood pressure >140 mmHg or diastolic blood pressure >90 mmHg; Diabetes mellitus= physician diagnosis of diabetes or fasting plasma glucose > 126 mg/dl.

* Among ever smokers

† Among current smokers
