# Supplementary material for: The Association of Systemic Microvascular Changes with Lung Function and Lung Density: A Cross-Sectional Study
Source: PLoS One. 2012 Dec 20;7(12):e50224. doi: 10.1371/journal.pone.0050224 (PMC3527439; doi:10.1371/journal.pone.0050224)
Supplement: Table S2 — Lung function and percent low attenuation area (%LAA) by retinal vascular caliber as measured by central retinal artery equivalent (CRAE). (DOCX) [file pone.0050224.s004.docx]

**Table S2. Lung function and percent low attenuation area (%LAA) by retinal vascular caliber as measured by central retinal artery equivalent (CRAE).**

|  | **Quartile of Central Retinal Artery Equivalent** | | | | **Mean difference per 1 SD unit of CRAE (95% CI)** | **P-value** |
| --- | --- | --- | --- | --- | --- | --- |
|  | **1^st^ Quartile 78.9-134.9** | **2^nd^ Quartile 135.0-144.2** | **3^rd^ Quartile 144.3-152.9** | **4^th^ Quartile >153.0-195.5** |  |  |
|  | N=854 | N=855 | N=855 | N=855 | N=3419 |  |
| **FEV_1_,mL** |  |  |  |  |  |  |
| Model 1† | 10 | -16 | -16 | 0 | -15 (-29, 0.5) | 0.06 |
| Model 2‡ | 14 | -3 | 12 | 0 | -3 (-18,11) | 0.70 |
| Model 3§ | 15 | -9 | 14 | 0 | -4 (-18,11) | 0.60 |
| **FEV_1/_FVC,%** |  |  |  |  |  |  |
| Model 1† | -0.2 | -0.4 | -0.9 | 0 | -0.3 (-0.6,-0.0) | 0.03 |
| Model 2‡ | -0.1 | 0.2 | 0.4 | 0 | -0.1 (-0.4, 0.2) | 0.50 |
| Model 3§ | 0.1 | -0.2 | -0.2 | 0 | -0.1 (-0.3, 0.2) | 0.70 |
| **LAA, %** |  |  |  |  |  |  |
| Model 1† | -0.9 | -0.5 | -1.2 | 0 | -0.4 (-0.8, 0.0) | 0.07 |
| Model 2‡ | -0.9 | -0.2 | -0.7 | 0 | -0.2 (-0.6, 0.2) | 0.30 |
| Model 3§ | -1.0 | -0.2 | -0.6 | 0 | -0.2 (-0.6, 0.3) | 0.50 |

Abbreviations: SD=standard deviation; CRAE=Central Retinal Artery Equivalent; CI=confidence interval

† Model 1: Adjusted for age, gender, race/ethnicity, body mass index, height, waist and hip circumference and, for CT analyses, CT scanner type

‡ Model 2: Adjusted for all the variables in model 1 plus cigarette smoking status, cigarette pack years and urine cotinine

§ Model 3: Adjusted for all the variables in model 2 plus cigar-years, pipe-years, environmental tobacco exposure, occupational exposure to dust, asthma before age 45, family history of emphysema, chronic bronchitis, educational attainment, diabetes mellitus, fasting blood glucose, hypertension, systolic blood pressure, diastolic blood pressure, high-density lipoprotein, low-density lipoprotein, C reactive protein, fibrinogen, aspirin use, beta blocker use, angiotensin II receptor blocker and/or angiotensin converting enzyme inhibitor use, statin use, diuretic use, hormone replacement therapy use, bronchodilator use, oral or inhaled steroid use.
